# Personality matters: exploring the relationship between personality and stress physiology in captive African lions

**DOI:** 10.1186/s40850-022-00126-9

**Published:** 2022-06-02

**Authors:** Janice Vaz, Alana Bartley, John Hunt

**Affiliations:** School of Science, Western Sydney University, Locked Bag 1797, Penrith, NSW 2751 Australia

**Keywords:** Animal personality, Big cats, Coping style, Felids, Stress glucocorticoids, Welfare

## Abstract

**Background:**

Considering animals as individuals and not as species is becoming increasingly essential to animal welfare management in captive settings. Recent studies on big cat personalities and coping strategies suggest personality can help big cats cope in their surroundings. Yet a large portion of the published literature focuses on understanding either the personality or stress physiology of big cats. Our research shows how integrating an improved understanding of the personality of big cats with stress physiology may enhance welfare, especially for endangered species like African lions. By using a wild cat personality checklist, this study compared the key personality dimensions of 22 African lions with its faecal glucocorticoids and assessed factors influencing their personality and stress physiology.

**Results:**

We found two reliable personality dimensions for African lions (dominance and agreeableness) and identified key factors (sex, age and location) that may influence their personality. Further, on testing if these factors influenced the stress physiology through variations in glucocorticoid levels, there was no significant difference. However, there was a strong negative association between agreeableness and glucocorticoid levels. These results suggest that the behavioural traits loading positively and higher for agreeableness are associated with lower glucocorticoid stress levels, which may assist a lion to cope with stressors in its surroundings.

**Conclusions:**

Our findings highlight this integrated approach of linking personality and stress physiology of big cats can be beneficial for caretakers. For example, during stressful veterinary procedures or in reintroduction programs, recognizing the personality of lions can help in designing or providing them with resources that will alleviate stress. Thus, there is a need for more interdisciplinary approaches that will contribute towards enhancing the individual and overall welfare of big cats.

**Supplementary Information:**

The online version contains supplementary material available at 10.1186/s40850-022-00126-9.

## Background

Improving the individual and overall welfare of big cats is an ongoing concern [1]. Previously, zoos around the world managed big cats following a standard set of husbandry protocols. These standards may include the guidelines for carnivores set by each zoological regulator, such as the husbandry guidelines for lions [2]. Australia manages captive lions under open range zoos, standard zoos, circuses, and rescue centres. In captivity, the welfare of these lions is a considerable concern, as it is difficult to mimic their wide-ranging natural habitat [3]. The ‘one size fits all’ welfare strategy does not recognise personality traits and may not be suitable to address individual animals’ needs. Thus, more emphasis is being placed on understanding individual behavioural differences [4, 5]. Individual differences, or animal personality, is defined as the set of behaviours exhibited consistently across time and situations [6–8]. One way of understanding a big cat’s personality is by observing its behaviour or coping style under a challenging situation [7, 9].

A coping style comprises an external behavioural with an internal physiological stress response; this response is consistent over time and is characteristic to a certain group of individuals when faced with a stressor [7]. Internally, the animal initiates a neuroendocrine stress response when faced with a stressor, that releases stress hormones called catecholamines (rapid flight-fight response) and glucocorticoids (slow responding endocrine response) with acute or chronic effects [10–12]. However, the way an animal perceives these stressors may vary due to its personality, which is highlighted in its glucocorticoid (GC) levels [13]. Consistent individual variation in the stress physiology has been observed while measuring cortisol in the hypothalamic-pituitary–adrenal (HPA) axis and assessing cardiovascular activity in the sympathetic–adrenal–medullary (SAM) system [9, 14, 15]. This individual variation, particularly higher GC, has been linked to poor coping styles and may also be linked to maladaptive behavioural traits [16]. On the other hand, certain personality traits may help individual felids cope better to perceived stressors. An example is the tendency among clouded leopards to hide: this tendency demonstrates fearfulness and correlates with higher stress levels [17].

A variety of behavioural tests and checklists have been developed in the past decade to help identify the personalities among big cats; these tests and checklists have been used for, tigers (*Panthera tigris)* [18–20], jaguars (*Panthera onca)* [21], cheetahs (*Acinonyx jubatus)* [22–25], snow leopards (*Panthera uncia)* [26, 27], and in particular the Asiatic lion (*Panthera leo persica)* [28, 29] and African lion (*Panthera leo leo)* [30–32]. The multiple personality dimensions modified from human and primate studies on the Five-Factor Model (FFM) can be categorised as Openness to experience, Conscientiousness, Extraversion, Agreeableness and Neuroticism, abbreviated to OCEAN [33–36]. Previous studies on animals and wild felid personality have translated these human personality dimensions to suit feline behaviours to include dominance, which may not be very evident in humans [37, 38]. Similarly, studies have examined the stress physiology of felids and variation in GCs through cortisol or corticosterone in blood, nail, saliva, urine, faeces and hair [39–41].

Predominantly, a large portion of the published literature focuses on understanding either the personality or stress physiology of big cats. Of the seven studies that have focused on linking personality and stress in big cats, only one has studied this integrated relationship in African lions [42]. Their findings suggest that lions which were more social and less neurotic with unstable, nervous and negative affective traits, had lower GCs, indicating that more social animals coped better [42]. Previously, Gosling and John [43] discussed that extraversion, neuroticism, and agreeableness personality types were commonly seen across various species. However, many factors influence the relationship between personality and stress; these may be biological, social, environmental, life history and/or evolutionary traits, genetics and health [9, 32]. The influence of these dynamic factors may affect the animal, positively or negatively contributing to shaping its coping style. Investigating these factors specific to an individual lion may help to further understand the relationship between personality and stress.

This research explores the connections between big cats’ personality and individuals’ stress physiology to better understand individual animals’ coping capacity and vulnerability to stressors to promote wellbeing [7]. The study specifically investigates the influence of factors such as sex, age, core body temperature through the eye, origin and location, on the personality and stress physiology of the African lions. Based on past research [30], we predict males would be more dominant than females, and have different roles according to their age. Also, due to separate biological functions between the sexes seen among cubs, sub-adults and adults, there is likely to be a difference in the GC levels between the male and female lions, although there is currently little consensus over the direction of this sexual dimorphism [17, 44]. In addition, variation in the core eye temperature can be an indication of the perceived stressors, where higher eye temperatures are associated with higher stress levels and may also be linked with certain behavioural traits as seen among dogs, cats and various big cats [45–47]. Similarly, the origin of the lion (zoo bred or a circus lion), along with its current location (zoo or a rescue centre without visitors), may influence its coping style. Further, it was expected that personality differences would correlate with differences in GC levels. Lastly, we investigated if any of these nominated factors may also impact the integrated relationship of personality and stress physiology.

## Results

### Personality of African lions

#### Extracting personality axes

An examination of the total variance from unrotated Principal Component Analysis **(**PCA) indicated that four factors accounted for 80.53% of the variation in lion behaviour. However, parallel analysis indicated that only the eigenvalue of the first two principal components (PC) in the raw dataset exceeded these chance values, suggesting that these factors underlie the personality types (Table S3). Thus, the parallel analysis reduced the 4 components to 2 components explaining 62.99% variation and were labelled as PC1 and PC2, where a value of 0.4 or above was considered to be biologically important [32] (Table 1).

**Table 1:** Unrotated principal component analysis of behavioural traits in African lions. While the original PCA revealed 4 PCs with eigenvalues greater than 1, parallel analysis reduced this to the first two PCs - PC1 and PC2

	PC1 Dominance	PC2 Agreeableness
Eigenvalue	6.831	4.508
% Variance	37.950	25.043
Loadings		
Active	.245	**.794**
Affectionate	**−.702**	**.457**
Bold	**.500**	.380
Bullying	**.830**	.123
Clumsy	.238	**.476**
Defiant	**.653**	.296
Distractible	.225	**.837**
Erratic	**.902**	.111
Friendly to people	**−.666**	.539
Gentle	**−.922**	.122
Inquisitive	−.013	**.810**
Inventive	**.496**	**.668**
Irritable	**.858**	−.123
Playful	.074	**.884**
Solitary	**.484**	.110
Stable	**−.775**	.299
Trusting	**−.869**	.382
Vocal	.347	−.170

The boldface indicate values loading greater than 0.4 which are viewed as biologically important behaviours that contribute to the eigenvector

PC1 explained 37.95% of the cumulative variance in the data representing a dominance axis. The traits erratic, bullying, defiant, irritable, bold, solitary and inventive loaded strongly and positively, while the traits gentle, trusting, stable, affectionate and friendly to people loaded negatively. Hence, lions having higher PC1 scores were bolder compared to those with lesser scores, indicating more dominant individuals.

PC2 explained 25.04% of the variance in the data representing an agreeableness axis. The traits of being playful, distractible, inquisitive, active, inventive, friendly to people, clumsy and affectionate loaded strongly and positively. Hence, animals with higher PC2 scores were more agreeable, and those that scored low were more antagonistic. Based on the pattern of factor loadings, the two PCs were labelled as dominance, and agreeableness, respectively [48].

#### Effect of sex, origin, location, age and core eye temperature on personality

African lions rated higher for dominance differed significantly with sex; with males (*Mean* = 0.43, *SD* = 1.05) being significantly more dominant than females (*Mean* = − 0.62, *SD* = 0.45) (Table 2). In contrast, male (*Mean* = 0.03, *SD* = 0.86) and female lions (*Mean* = − 0.04, *SD* = 1.22) did not differ for agreeableness.

**Table 2:** ANOVA results comparing the effects of sex, lion origin and lion location on personality

Personality type	Factors	SS	*df*	*F*	*P*
Dominance	Sex	6.008	1,20	8.016	**0.010**
	Origin	0.442	1,20	0.430	0.519
	Location	0.408	1,20	0.396	0.536
Agreeableness	Sex	0.036	1,20	0.034	0.856
	Origin	1.427	1,20	1.458	0.241
	Location	3.838	1,20	4.472	**0.047**

The boldface values are significant at *P* = 0.05

Origin of the lion did not differ significantly for dominance or agreeableness. Lions who were rated higher on agreeableness varied significantly between the location 1 – Zambi Wildlife Retreat (ZWR) (*Mean* = − 0.19, *SD* = 0.91) and location 2 – Sydney Zoo (SZ) (*Mean* = 0.88, *SD* = 0.98), but not for dominance. Simple linear regression showed a negative relationship between dominance and age, with an *R*^*2*^ of 0.136, but it was not significant (Table 3). However, there was a significant negative relationship between agreeableness and age, with an *R*^*2*^ of 0.342 indicating that agreeableness declines with age. Further, there was no significant relationship between core eye temperature and dominance or agreeableness.

**Table 3:** Linear regression equation model to explore the relationship between age and core eye temperature on the intensity of personality types in captive African lions

Personality type	Factors	Std Coefficients		***t***	***df***	***F***	***P***
		Beta	Std. Error				
**Dominance**	Age	−.369	1.029	−1.777	1,20	3.157	0.091
	Core Eye Temperature	0.082	0.193	0.367	1,20	0.135	0.717
**Agreeableness**	Age	−.585	0.898	−3.222	1,20	10.381	**0.004**
	Core Eye Temperature	0.285	0.185	0.135	1,20	1.774	0.198

The boldface values are significant at *P* = 0.05

### Stress glucocorticoid hormones

#### Cortisol levels of lions

The faecal GC concentrations ranged from 0.18 ng/g to 0.21 ng/g among the lions, with an overall mean of 0.20 ± 0.007 ng/g (Table 4).

**Table 4:** Average cortisol levels (ng/g) for lions at both study sites

Lion	Average Cortisol (ng/g)
Males	0.202
Females	0.197
Study Site 1	0.201
Study Site 2	0.195

#### Effect of sex, origin, location, age and core eye temperature on stress physiology

Cortisol levels did not differ between sexes (*F*_*(1, 20)*_ = 2.659, *P* = 0.119), with no individual difference between the males (*Mean* = 0.202, *SD* = 0.007) and females (*Mean* = 0.197, *SD* = 0.007). Similarly, there was no significant difference in cortisol with the origin of the lion (*F*_*(1, 20)*_ = 1.128, *P* = 0.301), as the circus born African lions (*Mean* = 0.203, *SD* = 0.002) did not differ in their cortisol levels from zoo individuals (*Mean* = 0.199, *SD* = 0.008). In addition, there was no significant difference in cortisol among lions in the two locations (*F*_*(1, 20)*_ = 2.092, *P* = 0.164), suggesting that the lions from SZ (*Mean* = 0.195, *SD* = 0.010) did not differ in their cortisol levels from ZWR (*Mean* = 0.201, *SD* = 0.007). Simple linear regression showed no relationship between cortisol and age (*R*^*2*^ = 0.071, *F*_*(1, 20)*_ = 1.529, *P* = 0.231) nor between cortisol and core eye temperature (*R*^*2*^ = 0.001, *F*_*(1, 20)*_ = 0.019, *P* = 0.892).

### Relationship between personality types and stress

**Fig. 1:**
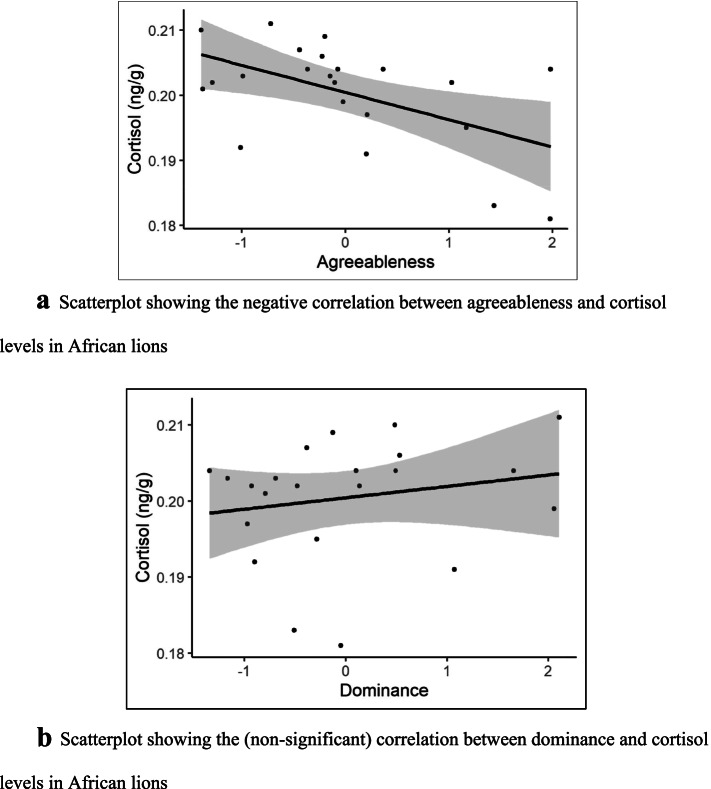
**a** Scatterplot showing the negative correlation between agreeableness and cortisol levels in African lions. **b** Scatterplot showing the (non-significant) correlation between dominance and cortisol levels in African lions

There was a strong negative correlation (*r* = − 0.533, *P* = 0.011) found between agreeableness and cortisol levels. In contrast, there was no significant relationship between dominance and cortisol levels (*r* = 0.196, *P* = 0.383) (Fig. 1a and b).

#### Effect of key factors on integrated relationship of personality and stress physiology

A partial correlation was conducted on controlling the effects of sex, but there was no relationship (*P* = 0.946) found between dominance and cortisol levels. However, when controlling the effects of age and location, there was still a strong relationship between agreeableness and cortisol levels (*P* = 0.036).

## Discussion

The present study assessed the links between the personality of African lions with their stress physiology to recognise factors that shape individual welfare. The personality of each lion was assessed by a rating method and the cortisol level was measured from fresh faecal samples [32, 40]. Further, our study quantifies the effects of factors such as sex, age, core eye temperature, origin and location on the personality of lions, followed by their stress physiology, and further on the integrated relationship of personality and stress. In short, we found two personality types among the studied African lions and found that sex, age and location of the lion may influence their personality. Further, on testing if these factors influenced the stress physiology through GC levels, there was no significant influence. However, there was a strong negative association between agreeableness and GC levels.

### Lion personality

The lions’ behaviour at both study sites were reliably rated by the keepers and the researcher. From the reliably rated behavioural traits, two significant components comprising of the dimensions - dominance and agreeableness were found for African lions, which were similar to results reported for various wild cats [32, 42, 49]. Dominance loaded positively and strongly for behavioural traits - erratic, bullying, defiant, irritable, bold, solitary and inventive, while the behavioural traits gentle, trusting, stable, affectionate and friendly to people loaded negatively. Similarly, previous studies rated reintroduced African lions for their boldness, another term commonly used for dominance [31] and Asiatic lions on a bold-shy axis for comparing individuals raised in captivity and others that were wild-rescued [28]. This suggests this dimension of dominance may be a prevalent trait among lions, and indicates that the social structure with roles of different individuals in a pride are important for their wellbeing [26]. For instance, from the behavioural trait loadings, the lions rated with high scores for dominance may want to compete and be the first to try everything, for example, in procuring food among others in a pride [50], whereas lions rated with low scores for dominance are usually submissive and may avoid confrontation with other dominant individuals [29]. These findings were also comparable to studies where African lions rated lower for dominance were found to cope by hiding [32, 42, 50].

The second dimension - agreeableness had the highest loadings for the behavioural traits playful, distractible, inquisitive, active, inventive, friendly to people, clumsy and affectionate, loading strongly and positively. This dimension of agreeableness, though not discussed in the past for African lions, has been applied to other wild cats such as clouded leopards [32] and domestic cats [51]. Agreeableness may also be required for members of the pride to get along with each other to lead a social life. Lions with high scores for agreeableness are likely to represent cats that are coping well and potentially serve as a source of enrichment for other cats [51]. In humans, exploring human personality has helped psychologists perceive the way people respond to stressors and have developed strategies to overcome them. Similarly, understanding the personality of lions can assist caretakers in ensuring their wellbeing by developing suitable approaches to cope with stressful circumstances such as veterinary procedures or reintroduction programs.

### Lion glucocorticoid levels

In the literature, the levels of GCs may vary among individuals and these variations seem to be influenced by the time of day, health status, age, sex, personality, body condition, time of year, stage of breeding and the environment [9, 52]. Thus, even among lions managed in the same setting or among related individuals, there may be intraspecific metabolic variations in the GC levels [17, 53–55]. In addition, other studies on African lions suggest that the variation found in GCs between individuals may act as markers to showcase the ongoing challenges faced by a lion [40, 56]. Thus, if the levels vary significantly and above the normal range of other individuals, it may reflect an imbalance [41]. Our results showed slight variations implying to animal individuality, but it did not vary significantly between individuals. The literature suggests that studies have adopted different hormone extraction methods to measure faecal cortisol levels in lions [9], where the average faecal cortisol ranged between 11.25–22.00 ng/g [56] and 0.12–0.24 μg/g [57]. Our study contributes towards the existing knowledge of the cortisol levels in captive African lions. Despite the different settings of the two study sites such as public exposure, the mean cortisol levels did not vary significantly, which supports the literature that without additional challenges, the lions may not perceive stressors or be engaged in a coping style.

### Factors influencing the personality dimensions and stress physiology of African lions

Our study assessed sex, age, core eye temperature, origin and location of the 22 lions to determine its influence on the personality types and stress physiology. On analysing the factors, we found that personality types were influenced by sex, age and location of the lion. Males were significantly more dominant than females. This describes the lions’ social structure of living in a pride in a harem-style composition, as also discussed by Gartner et al. [32]. The lesser extent of dominance among females could support their egalitarian behaviour for their own survival and in communal cub-raising because their reproduction depends on synchronous breeding and overall group territoriality [58]. Previous studies have also suggested that male lions are more aggressive than females [40], and it is also seen in other wild cats such as cheetahs where males scored significantly higher on the dominance and sociability dimensions than females [24]. Although previous studies found no significance of age on personality dimensions of African lions [32] or cheetahs [24], our results revealed that the younger individuals, more specifically lions aged between 3 and 7 years, were significantly more agreeable than older lions aged between 8 and 15 years. These variations may reflect the role of sub-adults and adults within pride behaviours [31]. For example, among reintroduced African lions, the sub-adults are more likely to be alert and active than adults [31]. In snow leopards, variance in curious/playful and active/vigilant was highest among mid-aged animals and lowest in older animals, which are similar to our findings. Also, the variance in calm/self-assured was highest in the youngest snow leopards and lowest in older animals, which describes the traits for agreeableness seen among lions of different age groups [27].

Secondly, lions at SZ (location 2) were rated higher for agreeableness than at ZWR (location 1). The small sample size at location 2 (*n = 4*) with younger aged individuals could result in these findings. Hence, a higher sample size or equal number of lions at both sites is essential to confirm this significance. Also, the limited sample size of having few rescued individuals from a circus versus those raised at a zoo, which varied across two locations (Table S1) could have resulted in such an outcome.

Contrary to the literature where males and females tend to vary in GC levels due to biological functions such as the differences in the amount of metabolites excreted, differences in plasma concentrations and differences in the structure [59], we did not find any difference in cortisol between the male and the female lions. However, past big cats studies also do not have a consensus and sometimes have shown either males or females having higher GCs such as African lions [41] Sumatran tigers [44] and North American clouded leopards [17]. It is possible that our results could be influenced by the contraceptive implants in the studied females that could suppress the release of GCs and decrease the adrenal steroid output [60].

In addition, the cortisol levels did not differ with the age across the two study periods. Similar findings were previously suggested for male African lions, where the concentrations of GCs were similar across age groups and did not vary with season [57]. The cortisol levels also did not vary with the other factors – location, core eye temperature or origin, which is likely due to the unequal sample size as seen in personality results above.

### Linking personality with stress physiology

Building an understanding of the connections between personality and stress physiology in African lions may help enhance their management and wellbeing. In this study, between personality types and stress levels, there was a negative relationship between agreeableness and cortisol levels, with more agreeable lions having lower cortisol levels. This reveals that agreeable individuals may overcome challenges better than other individuals who are antagonistic. Lions rated on agreeableness were engaged in more playful behaviours and show other carefree traits such as distractible, inquisitive, active, inventive, friendly to people which may also help them to get along with other members of the pride. They may also perceive stressors differently and hence reflect lower cortisol levels as compared to other individuals in the pride, and which is seen among other species too [61, 62]. These traits may help them to cope with a challenging situation. Although limited information is available on the integrated relationship of agreeableness and cortisol levels in human studies [63, 64], there were similar findings suggesting that agreeableness may contribute to a reduced HPA-axis response in a real-life interpersonal conflict [65].

Conversely, low scores for agreeableness may reflect poor socialisation and frustration [40]; these traits may be related to underlying health conditions as found among rescued domestic cats [66] and having higher cortisol levels [67]. Previously, Ones et al. identified agreeableness correlates weakly with Extraversion, is negatively related to Neuroticism and somewhat positively correlated to Conscientiousness [68].

#### Avenues for future research

Although there are many benefits of linking personality and stress, there is very limited work published on big cats taking this approach. Our study contributes to establishing this relationship for captive African lions. Being aware of a lion’s personality can help in caring for them more effectively and improving human-animal relationship. Maintaining a repository of the personality profiles of big cats’ can be valuable for big cats’ caretakers to enhance their knowledge of animals in their care and/or implement interventions such as veterinary assessments or enclosure developments. This information can also be useful for veterinarians to record health data.

We propose to record and store data on the personality and cortisol levels of big cats in the Zoological Information Management Software (ZIMS) that is accessible globally by ex-situ managers of zoos and rescue centres. Although it is expected that the reported GC concentrations would have resulted from different methodologies in sample collection, extraction and analysis, the storing of this data in a single online database will help to compare and contrast across the methods and further refine the technique beneficial for big cats. We also recommend conducting a biological validation before using commercial kits, which may be conducted by using samples from a naturally occurring stressful event, such as the introduction of a new individual to the group, or a translocation from one enclosure to another. The advantages of this study can then be applied to tailor animal welfare management specific to individual variation. For example, providing felids rated high on agreeableness with good hiding spots could reduce the impact of stressors, as seen among cheetahs rated on tense-fearful scores or among jungle cats with lower corticosterone levels [25, 69]. In addition, a “less agreeable” cat with higher GC levels may need those hiding spots even more. Thus, this information is also beneficial in exhibit design, conservation reintroduction programs, species survival recovery plans to incorporate the needs while bringing a pride of social animals together.

## Conclusions

In this study, two personality dimensions – dominance and agreeableness were identified for African lions. We found sex, location and age strongly related to two personality types, emphasising the social organisation of lions where males and females of different age groups play an important role in the pride. We also found that lions rated higher for agreeableness had lower cortisol levels, highlighting that their behavioural traits help them in developing better coping strategies. The current study further suggests developing and incorporating a more systematic approach in the management of individual lions in zoos, rescue centres or in reintroduction programs. The authors recommend that big cat management can collate personality and stress-related endocrine data into the Zoological Information Management Software (ZIMS), so it is accessible to big cat caretakers around the world. This would assist in understanding the factors influencing personality and stress to help improve individual management and thus overall welfare for big cats.

## Methods

### Study sites and animals

Twenty-two African lions (13 males and 9 females) from two locations were studied between June–August 2018 and May–December 2019. There were eighteen lions (9 males, 9 females) at location 1 -Zambi Wildlife Retreat (ZWR) and four lions (4 males) location 2 - Sydney Zoo (SZ). Secondary demographic data about the lions such as sex, age and enclosure size were obtained from the study site records (Table S1). Zambi Wildlife Retreat is a retirement home for big cats from circuses, the entertainment industry or zoo breeding programs and is closed to visitors. Sydney Zoo is a newly opened zoo (2019), with lions relocated from another Australian zoo - Taronga Western Plains Zoo in Dubbo, NSW. Out of the 22 individuals, five geriatric lions had retired from a circus while others were raised in zoos. The age group of the lions was between 3 and 16 years (*Mean* = 9.1, *SD* = 4.9), and the lions were housed in enclosures that had an area ranging from 220 to 1500 sq.m. with conspecifics that were either male or females, except one solitary male whose sibling had passed away. To prevent unwanted breeding, all females were under a birth control program that involved the subcutaneous implantation of deslorelin acetate (Suprelorin® implant; Virbac).

### Personality assessment for captive African lions

#### Data collection

Wild cat personality questionnaires and focal animal observations were used to create the personality profiles of the lions [70, 71]. The questionnaire, comprising 52 behavioural traits, was used to rate the lion’s personality (Table S2). The traits were rated on a 7-point Likert scale, where 1 represents “not at all” and 7 represents “very much”, describing the degree to which a behaviour is seen in an animal. For consistency in rating the animals, a definition list of each of these behavioural traits was shared with the keepers along with the personality checklist [25, 32, 42, 72]. The lions were rated by five raters; four lion caretakers (two at each study site), and the researcher, who were all experienced in wild cat behaviour. The keeper ratings were based on their overall keeper interactions during daily animal care, veterinary procedures, and previous behavioural observations. These ratings were dependent on either experience with the animals or on existing knowledge of feline behaviours [29]. To reduce potential biases among keepers’ ratings towards their favourite felid, the researcher observed lion behaviour following focal sampling methods on three random days from morning to evening and later completed the personality questionnaire [29, 73].

#### Inter-rater reliability of Behavioural traits

The Intra-class Correlation Coefficients (ICC) were used to measure the reliability of different raters. The mean ratings of the five raters (k raters) were run in RStudio version 1.2.5033 (RStudio, Inc. Boston, MA) to determine the ICC (3, k) scores [74, 75]. Behavioural traits with ICC values lower than 0.75 and confidence intervals overlapping zero were excluded from further analysis, as they were deemed unreliable [75]. If a behavioural trait was excluded from one study site based on this definition, it was automatically excluded from the second study site to ensure that the same behavioural traits contributed to our definition of personality. Eighteen out of 52 behavioural traits passed Inter-rater Reliability testing across both study sites. The reliabilities of mean ratings ICC (3, k) ranged from 0.76 (trusting) to 0.99 (erratic) for lions at location 1 and 0.76 (clumsy) to 0.99 (vocal) for lions at location 2.

### Stress physiology assessment for captive African lions

#### Sample collection

Fresh faecal samples (< 2 days old) were identified and collected from individual lions during behavioural observations or cleaning routines, and a total of one to three faecal samples were collected per individual opportunistically (Table S1). These samples were collected only during the dry season as faecal samples remain stable for 5 days during the dry season, but for < 1 day during the wet season [76]. Each sample was labelled and stored temporarily at − 20 °C at the zoo for 1–2 days, and later transported on ice to the laboratory and placed in the − 80 °C freezer for longer storage until further analysis. Freezing samples without any chemical treatment at − 80 °C increases the recovery of glucocorticoids and was processed when all samples were collected [77]. It was ensured that samples were collected on random days without any stressful event affecting the animals by informing the keepers prior visitation to the zoo.

#### Hormone extraction and enzyme immunoassay (EIA)

The labelled frozen samples were then placed in a freeze-drier (Alpha 1–4 LD plus) for 48 hours to obtain a dried sample [78]. The dried sample was ground using a mortar and pestle and sieved to attain a homogenised powder. 0.2 g of this faecal powder was mixed with 2 mL of 90% ethanol and placed on an orbital shaker for 30 minutes. Samples were centrifuged for 15 minutes at 5000 rpm following the standard extraction protocol from Arbor Assays K003-H1W (DetectX®, Arbor Assays™). The supernatant obtained was stored, while the residue was discarded [79]. This supernatant solvent was then dried under nitrogen vapour (N_2_) in a fume-cupboard - Dynaflow GRP. Later, using 400 μL of assay buffer, the dried sample extract was reconstituted with 100 μL of absolute ethanol and vortexed for 30 seconds.

The commercial DetectX® Cortisol Enzyme Immunoassay Kit K003-H1W (96 well plate) from Arbor Assays was used to analyse the levels of faecal cortisol. Following the manufacturer’s instructions and previous studies on felids, the samples were processed [3, 80]. The plate map was used to map the layout of the samples, controls, and standards. The plate was read in a BIO-RAD iMark microplate reader at 450 nm. The final hormone concentration was calculated by multiplying the pg/mL hormone concentration with the final extract volume (0.5 mL) and dividing the faecal sample mass (0.2 g) to derive the final faecal cortisol concentration in ng/g of sample. An average of these values per lion was used for further analysis.

#### Assay validation for lion faecal samples

Since big cats such as African lions are classified as Vulnerable in the IUCN Red List Assessment [81], acquiring permission to manipulate stress in the study animals would not be permitted, especially for the retired lions due to welfare concerns. The Arbor Assays DetectX Cortisol EIA Kit has been tested and validated for various species such as Amur tiger, giraffe, kudu, Reeve’s muntjac, white-handed gibbon, white rhino, zebra, and lion by the manufacturer in their product protocol (DetectX®, Arbor Assays™). Therefore, we used the Arbor Assays EIA commercial kit that was already tested on the faecal samples of lions to assay the lions in our study. The values we obtained line up with what was found in the protocol which ranged from 2.48 to 27.22 pg/mg. All faecal samples were assayed in duplicates and the sensitivity was reported as 27.6 pg/mL in the EIA Kit manual (product protocol, DetectX®, Arbor Assays™), while the limit of detection was 45.5 pg/mL. Further, our samples were within the linear range of the standard curve. These results indicate that EIA-K003-H5 kit is an analytically reliable assay for measuring cortisol concentrations in faeces of lions. The kit provides a mouse monoclonal antibody specific for cortisol to be detected in multi-species (DetectX®, Arbor Assays™). It also presents cross-reactivity with dexamethasone (18.8%), prednisolone (1-Dehydrocortisol) (7.8%), corticosterone (1.2%), cortisone (1.2%), progesterone (< 0.1%), estradiol (< 0.1%), cortisol 21-glucuronide (< 0.1%), 1α-hydroxycorticosterone (< 0.1%) and testosterone (< 0.1%). The repeatability or intra-assay variation between these duplicates were measured in RStudio version 1.2.5033 (RStudio, Inc. Boston, MA) using the ICC repeatability test (ICC = 0.77, 95% CIs = 0.67, 0.87). However, since samples were run opportunistically; running samples across multiple kits was not possible and inter-assay variation could not be calculated. Further, parallel displacements were carried out between standard and sample hormones, to detect the relationship between predicted and test samples [82]. A standard curve was plotted from synthetic CORT stock provided in the kit against its serial dilution. The samples were assayed in duplicates, with the mean of the two results being presented. To analyse if there was a significant relationship in the percentage of antibody bound between the standard curve and serial dilutions, a linear regression analysis was used [44]. The recovery of exogenous cortisol was added to the lion samples to analyse the efficiency of the faecal extracts (*R*^*2*^ = 0.9561) (Fig. S1).

#### Investigating the factors influencing personality and stress physiology

Information on the sex, age, origin and location of the lions was obtained from zoo records (Table S1). To assess the core eye temperature, an infrared thermal (IRT) imaging camera -FLIR T530 was used. Thermographic core measurements were used to measure the temperature (°C) in the lacrimal caruncle of each eye [45]. Images of the focal lion were captured by standing at a distance of approximately 3–6 m to avoid any disturbance to the animals. The thermal images were uploaded in the FLIR Tools software to assess the core eye temperature by pointing to the hottest area around the eye [83]. The lions were observed every hour on observation days to ascertain each animal’s average eye temperature.

### Statistical analysis

#### Extracting the principal components and determining personality dimensions

For the 18 behavioural traits that passed the ICC reliability test, we used Principal Component Analysis (PCA) in IBM SPSS version 27.0 (SPSS Inc., Armonk, NY, USA) to determine the significant eigenvectors of personality. PCA reduces the dimensions by combining the original behavioural traits into a reduced number of orthogonal eigenvectors to represent the maximum variability of the covariance structure of the data [84]. We considered eigenvectors as being significant if the associated eigenvalues were greater than 1 [32, 51] and eigenvectors were extracted based on the correlation (not covariance) matrix. The unrotated PCA indicated that 4 factors accounted for 80.53% of the variation in lion behaviour. However, we ran a parallel analysis that identifies factors having eigenvalues higher than values which may occur through chance, that reduced the significant eigenvectors extracted from the PCA to define our dimensions of personality in SPSS [32, 85–87] (Table S3). The parallel analysis reduced the 4 components to 2 components explaining 62.99% variation (Table 1). Individual behaviours that had factors loading greater than 0.4 were viewed as biologically important behaviours that contribute to that eigenvector [32]. The feline personality dimensions were then determined either by assessing the continuum of one personality dimension such as bold-shy [28] and by assessing multiple dimensions [32, 51]. This study used the multiple dimensions of the FFM and included dominance for wild cats to ensure consistency in assessing big cat personality [37, 38].

#### Determining the effects of sex, age, core eye temperature, origin, and location on personality and stress physiology

We used a one-way analysis of variance (ANOVA) to examine the effects of sex, origin, and location on the PCA dimensions of African lions and the stress physiology in SPSS. The level of significance, α, was set at 0.05. The PCA dimensions and cortisol levels were set as the dependent factor and sex, origin, and location as independent. In addition, linear regression was used to determine the relationship between PCA dimensions/cortisol and the age and core eye temperature of the lions.

#### Linking personality types with stress physiology and identifying factors influencing the integrated relationship

The resulting PCA personality scores were further used in investigating the relationship between PCA dimensions and cortisol levels using a Pearson’s correlation. To identify the effects of the significant factors affecting this integrated relationship, a partial correlation was used, where the key factors were treated as controlling variables when examining the relationship between personality and stress. Figures were constructed using the “ggplot2” package in RStudio version 1.2.5033 (RStudio, Inc. Boston, MA).

## Supplementary Information

**Additional file 1.**

## Data Availability

The datasets used and analysed during the current study are available from the corresponding author on request.

## Abbreviations

ACTH: Adrenocorticotropic hormone
CI: Class Intervals
EIA: Enzyme immunoassay
FFM: Five-Factor Model
GC: Glucocorticoid
ICC: Intra-class Correlation Coefficients
IRT: Infrared Thermal
NSB: Non-Specific Binding
OCEAN: Openness to Experience, Conscientiousness, Extraversion, Agreeableness, Neuroticism
PC: Principal Components
PCA: Principal Component Analysis
SZ: Sydney Zoo
ZIMS: Zoological Information Management Software
ZWR: Zambi Wildlife Retreat
