# Supplementary material for: Personality matters: exploring the relationship between personality and stress physiology in captive African lions
Source: BMC Zool. 2022 Jun 2;7:30. doi: 10.1186/s40850-022-00126-9 (PMC10127340; doi:10.1186/s40850-022-00126-9)
Supplement: Supplementary file 1 — Additional file 1. [file 40850_2022_126_MOESM1_ESM.docx]

**Supplementary information**

**Table S1** Demography of lions studied at our two study sites

| **Study Site** | **Lion** | **Sex** | **Age** | **Weight**  **(kg)** | **Origin**  **(zoo/circus)** | **Distribution*** | **Faecal samples per lion** | **Average Cortisol**  **(ng/g)** | **Enclosure size**  **(sq.m.)** |
| --- | --- | --- | --- | --- | --- | --- | --- | --- | --- |
| 1 | 1 | Male | 7 | 220 | Zoo | Pride | 3 | 0.199 | 900 |
|  | 2 | Female | 16 | 160 | Zoo | Pride | 2 | 0.203 | 900 |
|  | 3 | Female | 16 | 160 | Zoo | Pride | 1 | 0.192 | 900 |
|  | 4 | Male | 16 | 180 | Zoo | Pair | 2 | 0.209 | 300 |
|  | 5 | Female | 11 | 160 | Zoo | Pair | 3 | 0.195 | 300 |
|  | 6 | Male | 5 | 210 | Zoo | Pride | 3 | 0.204 | 900 |
|  | 7 | Female | 6 | 160 | Zoo | Pride | 3 | 0.197 | 900 |
|  | 8 | Female | 6 | 160 | Zoo | Pride | 2 | 0.202 | 900 |
|  | 9 | Female | 5 | 160 | Zoo | Pride | 2 | 0.181 | 900 |
|  | 10 | Male | 11 | 180 | Zoo | Pride | 2 | 0.21 | 900 |
|  | 11 | Female | 11 | 160 | Zoo | Pride | 3 | 0.201 | 900 |
|  | 12 | Male | 5 | 170 | Zoo | Pride | 1 | 0.211 | 900 |
|  | 13 | Male | 12 | 170 | Circus | Bachelor | 2 | 0.207 | 400 |
|  | 14 | Male | 12 | 170 | Circus | Bachelor | 2 | 0.204 | 400 |
|  | 15 | Male | 5 | 160 | Zoo | Solitary | 3 | 0.206 | 300 |
|  | 16 | Female | 15 | 160 | Circus | Pride | 1 | 0.202 | 220 |
|  | 17 | Male | 15 | 180 | Circus | Pride | 2 | 0.202 | 220 |
|  | 18 | Female | 15 | 180 | Circus | Pride | 1 | 0.203 | 220 |
| 2 | 19 | Male | 3 | 217 | Zoo | Bachelor | 1 | 0.191 | 1500 |
|  | 20 | Male | 3 | 197 | Zoo | Bachelor | 3 | 0.204 | 1500 |
|  | 21 | Male | 3 | 202 | Zoo | Bachelor | 3 | 0.204 | 1500 |
|  | 22 | Male | 3 | 230 | Zoo | Bachelor | 1 | 0.183 | 1500 |

*Distribution indicates animals living as solitary, pair, male bachelor group or pride

**Table S2**:

The Intra-class Correlation Coefficients (ICC) were used to measure the reliability of different raters. Thirty-four unreliable behavioural traits with ICC scores lower than 0.75 and Class Intervals (CI) overlapping 0 were excluded, and 18 behavioural traits passed the reliability test (in bold).

| Traits | Location 1 | | | Location 2 | | |
| --- | --- | --- | --- | --- | --- | --- |
|  | **ICC (3,k)** | **Lower CI** | **Upper CI** | **ICC (3,k)** | **Lower CI** | **Upper CI** |
| Active | **0.8** | **0.61** | **0.91** | **0.95** | **0.74** | **0.99** |
| Affectionate | **0.92** | **0.85** | **0.96** | **0.89** | **0.49** | **0.99** |
| Aggressive to conspecifics | 0.72 | 0.46 | 0.87 | 0.8 | 0.066 | 0.98 |
| Aggressive to people | 0.72 | 0.46 | 0.87 | 0.94 | 0.7 | 0.99 |
| Aimless | 0.7 | 0.421 | 0.86 | 0.89 | 0.471 | 0.99 |
| Anxious | 0.43 | -0.108 | 0.73 | 0.95 | 0.78 | 0.99 |
| Bold | **0.82** | **0.65** | **0.91** | **0.81** | **0.073** | **0.98** |
| Bullying | **0.85** | **0.71** | **0.93** | **0.88** | **0.41** | **0.99** |
| Calm | 0.63 | 0.29 | 0.83 | 0.8 | 0.039 | 0.98 |
| Clumsy | **0.88** | **0.77** | **0.95** | **0.76** | **-0.1266** | **0.97** |
| Constrained | 0.67 | 0.356 | 0.84 | 0.72 | -0.332 | 0.97 |
| Cool | 0.75 | 0.51 | 0.88 | 0.92 | 0.6 | 0.99 |
| Cooperative | 0.65 | 0.325 | 0.84 | 0.79 | 0.0068 | 0.98 |
| Curious | 0.54 | 0.036 | 0.78 | 0.78 | -0.0273 | 0.98 |
| Decisive | 0.58 | 0.181 | 0.8 | 0.83 | 0.168 | 0.98 |
| Defiant | **0.85** | **0.7** | **0.93** | **0.88** | **0.44** | **0.99** |
| Deliberate | 0.79 | 0.6 | 0.9 | 0.7 | -0.437 | 0.97 |
| Distractible | **0.85** | **0.71** | **0.93** | **0.88** | **0.45** | **0.99** |
| Dominant | 0.87 | 0.74 | 0.94 | 0.72 | -0.338 | 0.97 |
| Eccentric | 0.83 | 0.67 | 0.92 | 0.68 | -0.529 | 0.96 |
| Erratic | **0.99** | **0.97** | **0.99** | **0.94** | **0.73** | **0.99** |
| Excitable | 0.88 | 0.76 | 0.94 | 0.73 | -0.297 | 0.97 |
| Fearful of conspecific | 0.63 | 0.279 | 0.82 | 0.6 | -0.9 | 0.96 |
| Fearful of People | 0.308 | -0.337 | 0.67 | 0.99 | 0.93 | 1 |
| Friendly to Conspecifics | 0.79 | 0.59 | 0.9 | 0.75 | -0.189 | 0.97 |
| Friendly to People | **0.91** | **0.83** | **0.96** | **0.89** | **0.47** | **0.99** |
| Gentle | **0.86** | **0.73** | **0.93** | **0.92** | **0.61** | **0.99** |
| Impulsive | 0.81 | 0.63 | 0.91 | 0.651 | -0.662 | 0.96 |
| Independent | 0.64 | 0.302 | 0.83 | 0.95 | 0.77 | 0.99 |
| Individualistic | 0.79 | 0.6 | 0.9 | 0.75 | -0.189 | 0.97 |
| Inquisitive | **0.82** | **0.65** | **0.91** | **0.88** | **0.415** | **0.99** |
| Insecure | 0.484 | 0.0032 | 0.76 | 0.93 | 0.66 | 0.99 |
| Inventive | **0.79** | **0.59** | **0.9** | **0.94** | **0.72** | **0.99** |
| Irritable | **0.89** | **0.79** | **0.95** | **0.89** | **0.46** | **0.99** |
| Jealous | 0.81 | 0.64 | 0.91 | 0.68 | -0.503 | 0.96 |
| Persevering | 0.69 | 0.4 | 0.85 | 0.67 | -0.586 | 0.96 |
| Playful | **0.9** | **0.8** | **0.95** | **0.86** | **0.347** | **0.98** |
| Predictable | 0.71 | 0.438 | 0.86 | 0.65 | -0.66 | 0.96 |
| Quitting | 0.66 | 0.348 | 0.84 | 0.75 | -0.189 | 0.97 |
| Reckless | 0.88 | 0.76 | 0.94 | 0.68 | -0.508 | 0.96 |
| Self-assured | 0.426 | -0.111 | 0.73 | 0.66 | -0.61 | 0.96 |
| Smart | 0.7 | 0.41 | 0.86 | 0.87 | 0.37 | 0.99 |
| Solitary | **0.9** | **0.8** | **0.95** | **0.83** | **0.192** | **0.98** |
| Stable | **0.91** | **0.83** | **0.96** | **0.8** | **0.049** | **0.98** |
| Stingy | 0.84 | 0.69 | 0.92 | 0.74 | -0.232 | 0.97 |
| Submissive | 0.79 | 0.6 | 0.9 | 0.74 | -0.235 | 0.97 |
| Suspicious | 0.65 | 0.325 | 0.84 | 0.66 | -0.605 | 0.96 |
| Tense | 0.62 | 0.256 | 0.82 | 0.93 | 0.68 | 0.99 |
| Timid | 0.61 | 0.242 | 0.82 | 0.63 | -0.74 | 0.96 |
| Trusting | **0.76** | **0.54** | **0.89** | **0.91** | **0.55** | **0.99** |
| Vigilant | 0.55 | 0.122 | 0.79 | 0.9 | 0.539 | 0.99 |
| Vocal | **0.82** | **0.65** | **0.92** | **0.99** | **0.95** | **1** |

**Fig. S1** Regression plot for recovery of cortisol standard in the extract pool (extraction efficiency = 95%)

**Table S3.** Parallel analysis showing the reduction of four Principal Components (PCs) from our unrotated PCA to two PCs. PCs are considered significant (in bold) in parallel analysis when the raw data for an eigenvector (root) exceeds the mean value for that eigenvector.

| Root Raw Data Means Percentile | Significant Principal Components |
| --- | --- |
| 1.000000 6.831026 3.057763 3.505020 | PC1 - Dominance |
| 2.000000 4.507664 2.536895 2.849656 | PC2 - Agreeableness |
| 3.000000 1.728087 2.165428 2.400144 |  |
| 4.000000 1.429173 1.858596 2.072662 |  |
| 5.000000 .770636 1.592165 1.778667 |  |
| 6.000000 .637314 1.365494 1.529774 |  |
| 7.000000 .521530 1.160405 1.325590 |  |
| 8.000000 .337120 .975040 1.124383 |  |
| 9.000000 .313899 .816704 .950049 |  |
| 10.000000 .275559 .664730 .780072 |  |
| 11.000000 .241434 .531960 .649359 |  |
| 12.000000 .147397 .417716 .520334 |  |
| 13.000000 .112362 .313611 .405348 |  |
| 14.000000 .081452 .226160 .305640 |  |
| 15.000000 .025341 .153514 .219688 |  |
| 16.000000 .022564 .095675 .149537 |  |
| 17.000000 .013061 .050086 .091543 |  |
| 18.000000 .004380 .018057 .040050 |  |
